# A Bioinformatics Perspective on the Dysregulation of Ferroptosis and Ferroptosis-related Immune Cell Infiltration in Alzheimer's Disease

**DOI:** 10.7150/ijms.76660

**Published:** 2022-10-24

**Authors:** Lusi Zhang, Jia Fang, Zhenchu Tang, Yingying Luo

**Affiliations:** 1 Department of Ophthalmology, The Second Xiangya Hospital, Central South University, Changsha, Hunan, China.; 2 Department of Neurology, The Second Xiangya Hospital, Central South University, Changsha, Hunan, China.

**Keywords:** Alzheimer's Disease, ferroptosis, immune cell infiltration, hippocampus

## Abstract

Alzheimer's disease (AD) is the most prevalent dementia worldwide, but its pathophysiology and molecular events remain unknown. Herein, we first analyzed the differential expression pattern of patients' AD hippocampus through gene expression array data from the GEO database. *Notch2nl*, *TGFB1I1*, and *LTF* were up-regulated in AD patients, while *ARPC1A*, *CHGB*, and *MPV17* down-regulated. Second, dysregulation of ferroptosis related genes was demonstrated from our data: *PCBP2* and *FTL* significantly up-significant in AD hippocampus, while *VDAC2*, *LPCAT3*, *GSS*, *ACSL4*, and *ACSL6* significantly down-regulated. The protein-protein interactions (PPI) network revealed that *FTL* was involved in iron metabolism and utilization, while *ACSL4* and *ACSL6* were involved in a polyunsaturated fatty acids metabolism network. Gene correlation analysis on differential expressed genes (DEGs) indicated that ferroptosis regulates a series of biological processes and pathways related to AD pathogenesis. Third, ferroptosis-related DEGs regulated the immune cell infiltration pattern in the AD hippocampus, characterized by decreased memory B cells, increased memory resting CD4^+^ T cells, memory activated CD4^+^ T cells, and resting NK cells. The altered expression of ferroptosis-related DEGs affected the infiltration of specific immune cell types. The model constructed by the seven ferroptosis-related differential genes may accurately predict the outcome of AD occurrence. Finally, qPCR validation on these ferroptosis-related DEGs in APPswe/PSEN1dE9 mice confirmed the dysregulated expression of *Pcbp2*,* FTL*,* GSS*,* and ACSL4* in the AD hippocampus and forebrain. In conclusion, our results supported the conception that the AD brain revealed dysregulated ferroptosis and immune cell infiltration.

## Introduction

Alzheimer's disease (AD) is the leading cause of dementia. Over 90% of AD cases have a late-onset, occurring in individuals older than 65 1. AD is characterized by gradual memory loss associated with progressive brain atrophy. The pathological AD hallmark is the accumulation of β-amyloid (Aβ) and neurofibrillary tangles of hyperphosphorylated tau. Several factors contributed to the disease onset. Genetic etiology has been reported to be AD cause. For example, pathogenic mutations on amyloid precursor protein (APP), presenilin 1 (PSEN1) and presenilin 2 (PSEN2) may lead to familial AD form 2. Genetic risk factors account for approximately 70% of the risk in late-onset AD patients, with the *APOE* gene, which has three variants, e2, e3, and e4, being the most significant risk factor 3.

AD pathogenesis has been thoroughly studied for decades. The most prevalent AD theory is the amyloid hypothesis: the APP cleavage product Aβ is produced by γ-secretase. The imbalance between Aβ production and Aβ clearance leads to fibrillar amyloid within amyloid plaques 3. Another crucial factor leading to AD is secondary tau pathology, which is associate with hippocampal synaptic impairments and memory deficits in animal models 4. Furthermore, TAR DNA-binding protein 43 (TDP-43) aggregates are recurrently found in AD 5. However, the complex AD pathophysiology remains unknown, and no effective AD treatments exists. Understanding the molecular events that lead to damage downstream of AD pathology holds great promise for developing new therapy.

Among the degeneration of the AD brain, deficits of neuronal death can be observed in the AD brain, including apoptosis 6, autophagy 7, necroptosis 8, and pyroptosis 9. Recent evidence suggests that ferroptosis, a novel defined form of iron-dependent regulated cell death (RCD), may play a role in AD pathogenesis 10, 11. Iron involves in maintaining normal brain functions, including mitochondrial respiration, myelin synthesis, and neurotransmitter synthesis or metabolism 11. In AD pathogenesis, iron is crucial in oxidative stress 12. Iron homeostasis is typically altered in AD patients, including lower serum iron levels 13 and iron overload in AD brain regions 14, 15. Iron plays a vital role in ferroptosis. Ferroptosis is usually triggered by phospholipid peroxidation, which is produced by a process involving iron transition, reactive oxygen species (ROS), and polyunsaturated fatty acid (PUFA) chains 16. During ferroptosis, the iron-dependent Fenton chain reaction is initiated and amplified by phospholipid hydroperoxides (PLOOHs), which react with ferrous and ferric ions to generate the free radicals PLO• and PLOO•. These free radicals, in turn, drive the destructive peroxidation chain reaction. Furthermore, iron participates in a series of redox-based metabolic processes that are contribute to the generation of cellular ROS 17. Overall, ferroptosis is considered a pathological AD feature. Ferroptosis modulation can serve as a therapeutic avenue. However, the precise mechanism of how ferroptosis induces neural death in the brain remains unclear and must be explored.

To unravel the molecular basis underlying the loss of neurons in a specific brain region in the AD context, we used the hippocampus gene expression array data of control and AD subjects to detect the possible mechanism and the effect of ferroptosis on AD. Our results demonstrated that the expression of several ferroptosis-related genes is dysregulated in the AD hippocampus, which mainly involves networks associated with iron metabolism, iron utilization and PUFA metabolism, and is highly coexpressed with other differentially expressed genes (DEGs). Furthermore, these genes alter the infiltration of specific types of immune cells in the AD hippocampus. Therefore, our findings suggest that ferroptosis plays an essential role in AD pathogenesis and that inhibiting ferroptosis could be a potential therapeutic target for AD treatment.

## Methods

### Data source

Hippocampus gene expression array data of control and AD subjects and corresponding clinical features were obtained from the Gene Expression Omnibus (GEO) database (https://www.ncbi.nlm.nih.gov/geo/) in the National Center of Biotechnology Information (NCBI) (https://www.ncbi.nlm.nih.gov/). Our study comprised data from 116 subjects (65 controls and 51 AD patients) derived from GEO Datasets GSE1297, GSE5281, and GSE48350.

Forty-one ferroptosis-related genes were obtained from the Kyoto Encyclopedia of Genes and Genomes (KEGG) pathway hsa04216 (https://www.kegg.jp/pathway/hsa04216. The gene list is as follow: *SLC3A2*, *SLC7A11*, *GCLC*, *GCLM*, *GSS*, *GPX4*, *ALOX15*, *LPCAT3*, *ACSL6*, *ACSL4*, *ACSL1*, *ACSL5*, *ACSL3*, *TP53*, *SAT2*, *SAT1*, *TFTFRC*, *STEAP3*, *SLC11A2*, *SLC39A8*, *SLC39A14*, *PCBP2*, *SLC40A1*, *CP*, *PCBP1*, *FTH1*, *FTL*, *MAP1LC3C*, *MAP1LC3B*, *MAP1LC3A*, *MAP1LC3B2*, *ATG5*, *ATG7*, *NCOA4*, *PRNP*, *HMOX1*, *VDAC2*, *VDAC3*, *CYBB*, and *FTMT*.

### Data combination, normalization, and differential expression analysis

To analyze publicly available data sets from different datasets, we processed expression data using the limma and sva packages in R 4.1.1, including data combine and batch effect removal. All datasets were then normalized using the R package preprocessCore. Differential expression analysis was performed using the R package limma. The threshold of DEGs was set as |log fold change (FC)| >1, *P* < 0.05. The R package ggplot2 was used to visualize the volcano plots and box plots of DEGs. Heatmaps were built using the R package pheatmap. The gene set used for further analysis was generated by surveying ferroptosis-related DEGs, revealing involvement of seven hub genes.

### Gene Ontology and KEGG Enrichment

Gene Ontology (GO) and KEGG pathway analyses were performed with the R package clusterProfiler for all DEGs as well as 7 ferroptosis related hub genes. *P* values < 0.05 was considered statistically significant.

### Protein-Protein Interaction Network Construction

To further explain the interactions between ferroptosis and AD pathology in the brain, we performed protein-protein interaction (PPI) network analysis was using the STRING database 18 (https://string-db.org/) on ferroptosis-related genes and our DEGs. A confidence score > 0.4 was considered significant. The PPI results were then imported into Cytoscape 3.9.1. The MCODE and Centiscape 2.2 plug-ins were used to identify out the hub genes in the network. Each network's top 5 or 10 genes were generated using the MCC algorithm with the Cytoscape plug-in cytoHubba.

### Assessment of immune cell infiltration

The R package CIBERSORT was used to evaluate the immune infiltration of 22 types of immune cells from previously obtained gene expression data. The R package ggplot2 was used to visualize the proportion of each immune cell subtypes and immune cell type with significantly differential infiltrate level (*P* < 0.05). The relationship between the expression of seven ferroptosis-related hub genes and infiltrating immune cells was displayed using the R package ggplot2.

### Gene Set Enrichment Analysis

Pairwise correlations between the seven ferroptosis-related hub genes were visualized as chord diagrams through the R package circlize. Pairwise correlations were also performed between the seven ferroptosis-related hub genes and all the DEGs we obtained from the public database. Gene set enrichment analysis (GSEA) was performed with default parameters through the GSEA software for the pairwise correlation results against the Reactome data set.

### Diagnostic receiver operating characteristic curve

The diagnostic ROC curve was drawn based on AD status in our 65 controls and 51 AD patients. The area under the curve (AUC) represents the probability of estimatng the accuracy of the actual observation rate and the predicted disease probability. Logistic regression models were implemented by using the R function glm. Our logistics model = 14.128 + (-0.4337 × GSS) + (1.5223 × PCBP2) + (-0.7593 × ACSL4) + (-0.8506 × FTL) + (-0.2677 × ACSL6) + (-0.7829 × LPCAT3) + (-1.6571 × VDAC2). The ROC analysis was performed with the R package pROC, and the data visualization was performed using R package ggplot2.

### qPCR validation of differentially expressed ferroptosis-related hub genes in the hippocampus and forebrain cortex of AD mice

The hippocampus and forebrain cortex tissue were obtained from 6-months-olds APPswe/PSEN1dE9 mice and C57BL/6 wildtype mice. This study was approved by the Ethics Committee of Xiangya Hospital, Central South University, China. The hippocampus and forebrain cortex were cut, homogenized, and stored at -80 °C. TRIzol RNA Isolation Reagents (Thermo Scientific™, Waltham, MA, USA) were added to the tissue homogenates, and RNA was extracted according to the manufacturer's protocol. Approximately 1 µg total RNA was used to reverse-transcript into cDNA with RevertAid First Strand cDNA Synthesis Kit (Thermo Scientific™, Waltham, MA, USA). Quantitive PCR was used to detect the expression of seven ferroptosis-related hub genes. The mouse transcripts were amplified using the following primers: 5'-AATCAATGCCAGGCTTTCCTC-3' and 5'-TTAAAACCTGGAATCGCTGACTG-3' for *Pcbp2*, 5'-CACCTACCTCTCTCTGGGCT-3' and 5'-CGCGATCGTTCTGAAACTCG -3' for *FTL*, 5'-AGAGAAGTGGAACACCGATAAC-3' and 5'-CAGCCCTCGTAACCAAAGACA-3' for *Vdac2*, 5'-GGCCTCTCAATTGCTTATTTCA-3' and 5'-AGCACGACACATAGCAAGGA-3' for *LPCAT3*, 5'- CTTCCTCTTAAGGCCGGGAC-3' and 5'-TGCCATAGCGTTTTTCTTAGATTT-3' for *ACSL4*, 5'-GCCCCATTCACGCTCTTCCCC-3' and 5'-ATGCCCGGCCTGCTTAGCTC-3' for *GSS*, 5'-AAGCAGTCGGAAGAAGTGGAG-3' and 5'-GGCATCGTCATAGTAATGGGTAAG-3' for *ACSL6*, 5'-CACGATGGAGGGGCCGGACTCATC-3' and 5'-TAAAGACCTCTATGCCAACACAGT-3' for β-actin. Quantitative real-time PCR was conducted using an Applied Biosystems™ StepOnePlus™ Real-Time PCR System (Roche, Inc., Waltham, MA, USA) with Roche FastStart™ Universal SYBR^®^ Green Master Mixes (Thermo Scientific, Inc., Basel, Switzerland). Each group contained tissue from three animals. Statistically significant differences between groups were determined using an unpaired *t* test.

## Results

### Transcriptome alteration is revealed within hippocampus samples of AD patients

**Figure 1:**
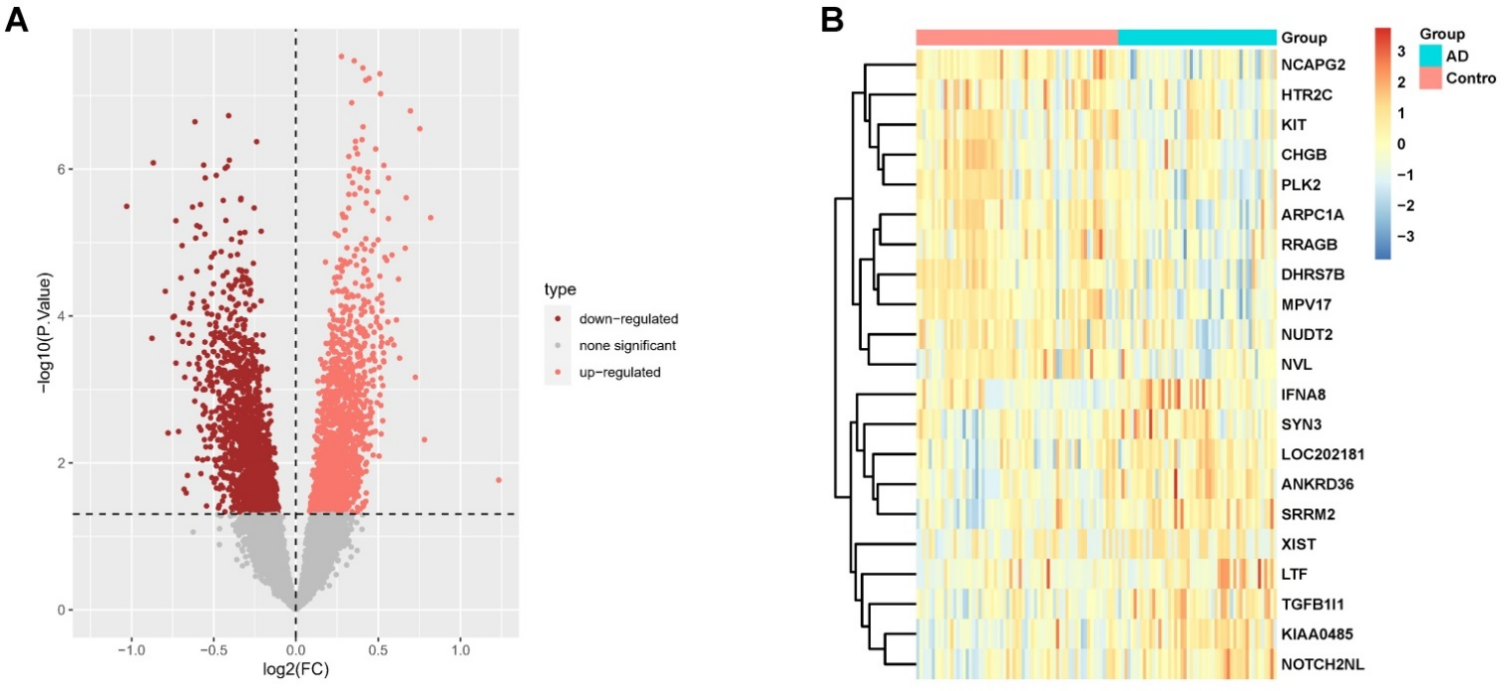
DEGs screening in AD hippocampus compared to normal controls. **(A, B)** Volcano plot (A) and heatmap (B) in datasets GSE1297, GSE5281, and GSE4835.

Our study incorporated 116 hippocampus gene expression array data from the GEO database including 65 control subjects and 51 AD subjects. Table 1 presents the basic information for these subjects. These data come from three separate datasets; batch effect removal and data normalization were initially conducted to combine them (Supplementary Fig. 1). There were 3331 DEGs in the AD hippocampus, where 1613 genes were down-regulated, and 1718 genes were up-regulated. The DEGs are shown in the volcano plot (Fig. 1A) and heatmap (Fig. 1B). The genes corresponding to the top 20 differences in transcriptome are listed in the heatmap. Among them, *Notch2nl*, *TGFB1I1*, and *LTF* were up-regulated in AD patients, while *ARPC1A*, *CHGB*, and *MPV17* were down-regulated.

### Enrichment Analysis of GO and KEGG pathways reveal some crucial processes and pathways in AD pathology

**Figure 2:**
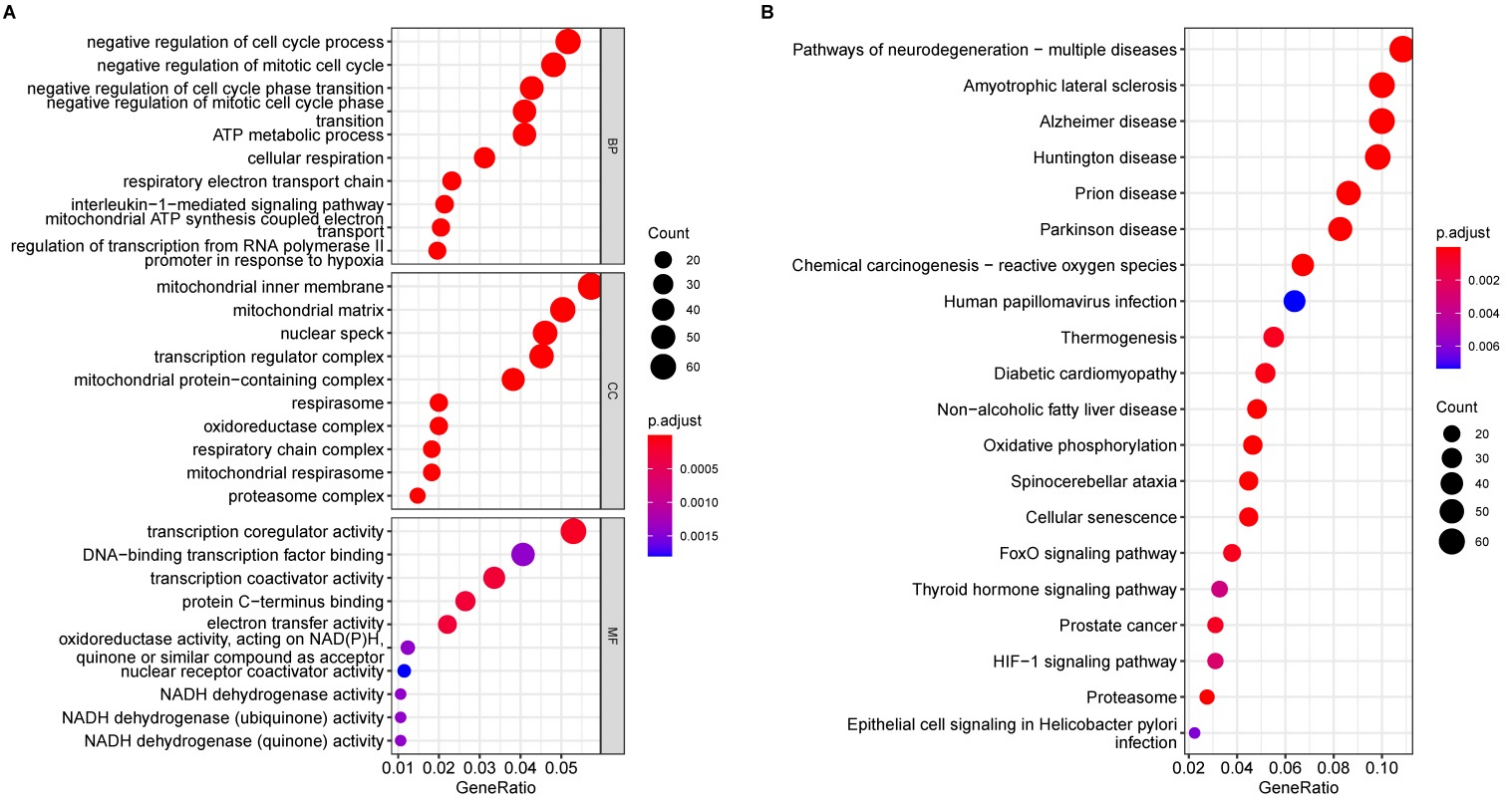
** Enrichment Analysis of GO and KEGG Pathways of DEGs in AD. (A)** Results of GO enrichment analysis of the DEGs. **(B)** The top 20 pathways result in GO enrichment analysis of the DEGs.

Functional enrichment analysis of the DEGs from the transcriptome analysis yielded statistically significant GO terms and KEGG pathways involved in AD pathology. In AD subjects, the top ten biological processes of the DEGs are mainly related to negative regulation of the cell cycle, ATP metabolic process, cellular respiration, and IL1β-mediated signal pathway (Fig. 2A), indicating that mitotic cell cycle and cell cycle transition, mitochondrial function and respiratory electron transport chain, and IL1β-related inflammation were all affected in AD. GO terms in cellular components associated with DEGs implicated in mitochondria matrix and inner membrane, respiratory chain complex, and proteasome complex (Fig. 2A), are crucial pathways in AD pathology. DEGs involve GO molecular function, including transcription coregulator/coactivator activity, NADH-related oxidoreductase activity, and electron transfer activity, which is significantly affected (Fig. 2A).

KEGG analysis of the DEGs revealed 38 significant enrichment pathways (P < 0.05) (Supplementary Table 1); Fig. 2B displays the top 20 pathways. Enriched AD categories are also involved in other neurodegenerative diseases, such as Huntington's disease, Prion disease, Spinocerebellar ataxia, and Amyotrophic lateral sclerosis. Moreover, other enriched pathways involve reactive oxygen species, oxidative phosphorylation, cellular senescence, and proteasomes.

### The expression of ferroptosis related genes is dysregulated in AD hippocampus samples

**Figure 3:**
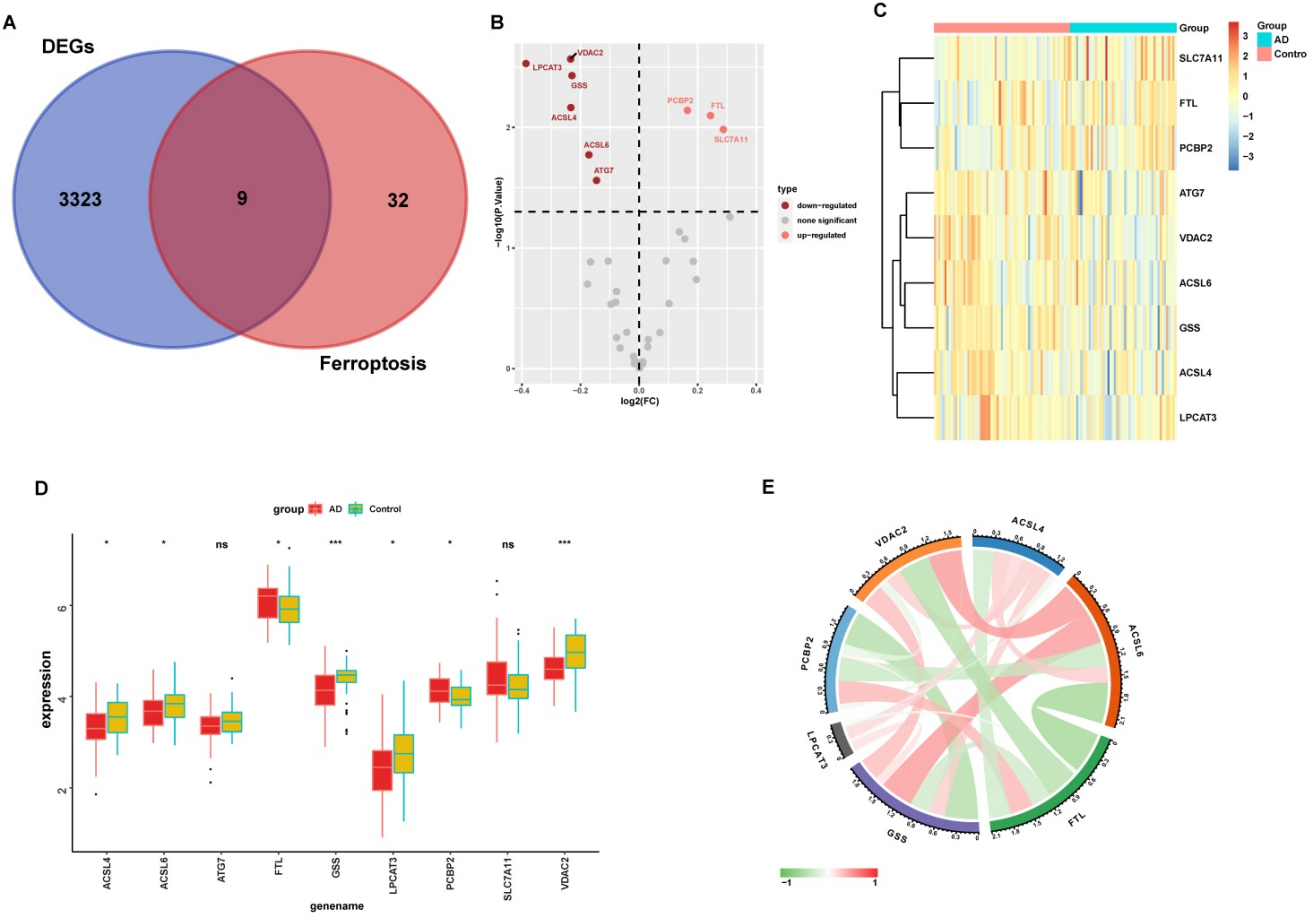
** Ferroptosis-related genes are dysregulated in the AD hippocampus. (A)** Venn diagram showing the overlapping genes of ferroptosis-related genes and DEGs. The expression of overlapping genes in the volcano plot **(B)**. Significant up-regulated and down-regulated genes are labeled as pink and dark red dots, respectively, in the volcano plot. **(C)** Heatmap displays the expression level of nine differential ferroptosis-related genes in AD and control groups. **(D)** The expression levels of the nine ferroptosis-related genes were visualized with a boxplot. *, *P* < 0.05; ***, *P* < 0.001; ns, not significant. **(E)** The correlations between DEGs. The color lines represent the correlation between gene expression. Red represents a positive correlation, while green represents a negative correlation. The dimmer the color or the thicker the line, the higher the correlation strength.

Emerging research between ferroptosis and neurodegenerative disease was reported in a recent study that suggested that ferroptosis contributes to AD pathology. To identify possible roles of ferroptosis-related genes in AD, we obtained the intersection of ferroptosis-related genes and our above DEGs (Fig. 3A), which yielded a subset of nine genes in the volcano plot (Fig. 3B). As evident in Figs 3B and 3C, *PCBP2*, *FTL*, and *SLC7A11* were significantly up-regulated in AD, while *VDAC2*, *LPCAT3*, *GSS*, *ACSL4*, *ACSL6*, and *ATG7* were down-regulated, indicating that ferroptosis was altered in AD hippocampus.

Furthermore, the expression levels of these nine ferroptosis-related genes were analyzed in these samples using boxplots (Fig. 3D), and seven ferroptosis-related genes were subsequently validated. *PCBP2* and *FTL* were significantly up-regulated in AD hippocampus samples compared with normal tissues, while *VDAC2*, *LPCAT3*, *GSS*, *ACSL4*, and *ACSL6* were significantly down-regulated. We used the circlize tool to determine the correlations between these seven significantly differential expressed genes. Strong positive correlations between these genes suggest a co-activation relationship between them. *FTL* and *ACSL6* exhibited a negative expression correlation, while *ACSL6* and *GSS* exhibited a positive expression correlation (Fig. 3E).

### PPI Network construction of ferroptosis related genes

**Figure 4:**
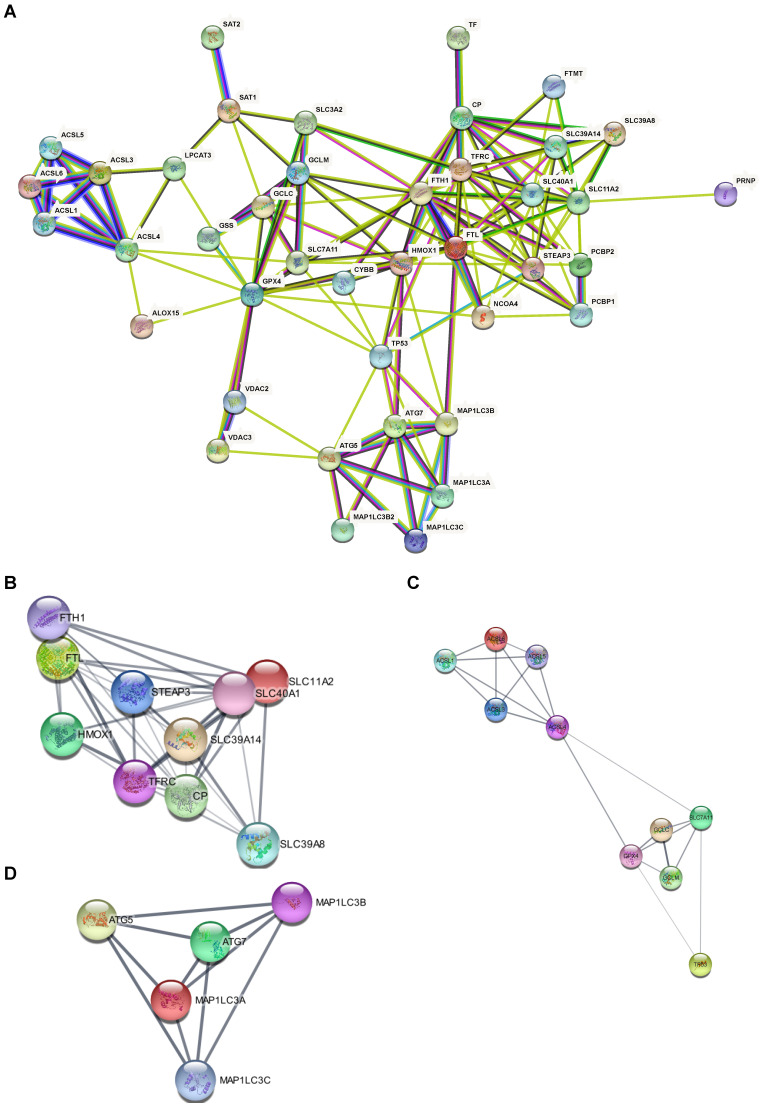
** PPI Network of ferroptosis related genes suggests that dysregulation of ferroptosis was correlated with AD. (A)** PPI Network of ferroptosis-related genes. **(B, C, D)** Subclusters of ferroptosis-related DEGs.

Based on the 41 ferroptosis-related genes and the PPI repository STRING, we constructed functional interaction between ferroptosis proteins (Fig. 4A). Our network demonstrated 42 nodes and 144 edges. Some distinct clusters with a high combined score were found among these ferroptosis-related genes (Table 2). The up-regulated expressed gene *FTL* in the AD hippocampus involved the network (Fig. 4B) which is associated with iron metabolism and utilization 19. Likewise, down-regulated expressed genes *ACSL4* and *ACSL6* were involved in another network (Fig. 4C) of PUFA metabolism 20, while *ATG7* was the hub gene of the third network (Fig. 4D). The PPI results suggest that dysregulation of ferroptosis is correlated with AD.

### Ferroptosis is correlated with AD

**Figure 5:**
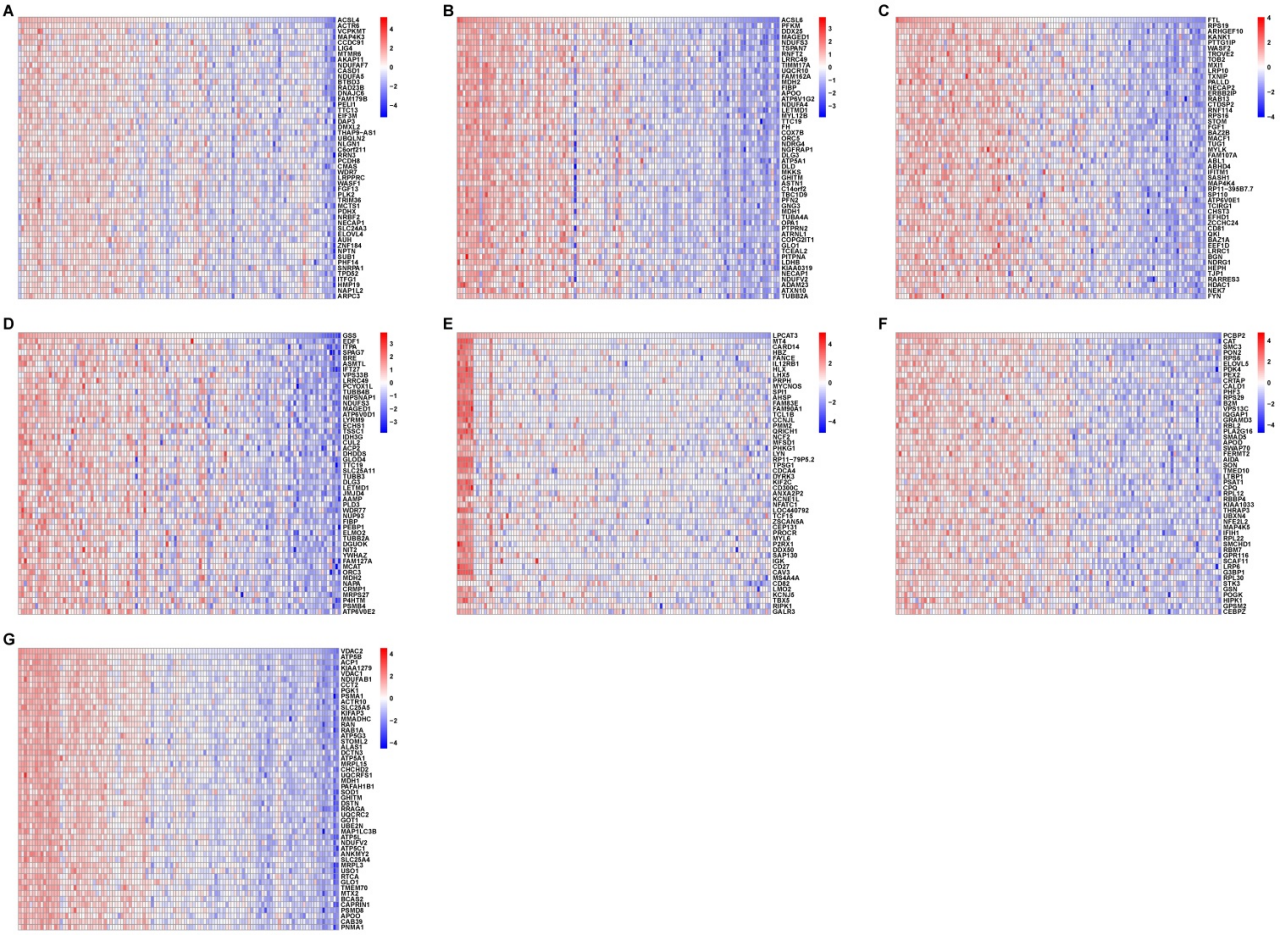
** Correlation between ferroptosis-related DEGs and AD DEGs. (A-G)** Heatmaps of the top 50 coexpressed genes to *ACSL4*, *ACSL6*, *FTL*, *GSS*, *LPCAT3*, *PCBP2*, and *VDAC2*.

To confirm the relationship between the seven ferroptosis-related hub genes and the 41 ferroptosis-related genes in the AD patients, we performed correlation analyses of these two sets of expression data across all controls and AD patients. The heatmaps in Fig. 5 revealed the top 50 coexpressed genes to each ferroptosis-related DEGs.

**Figure 6:**
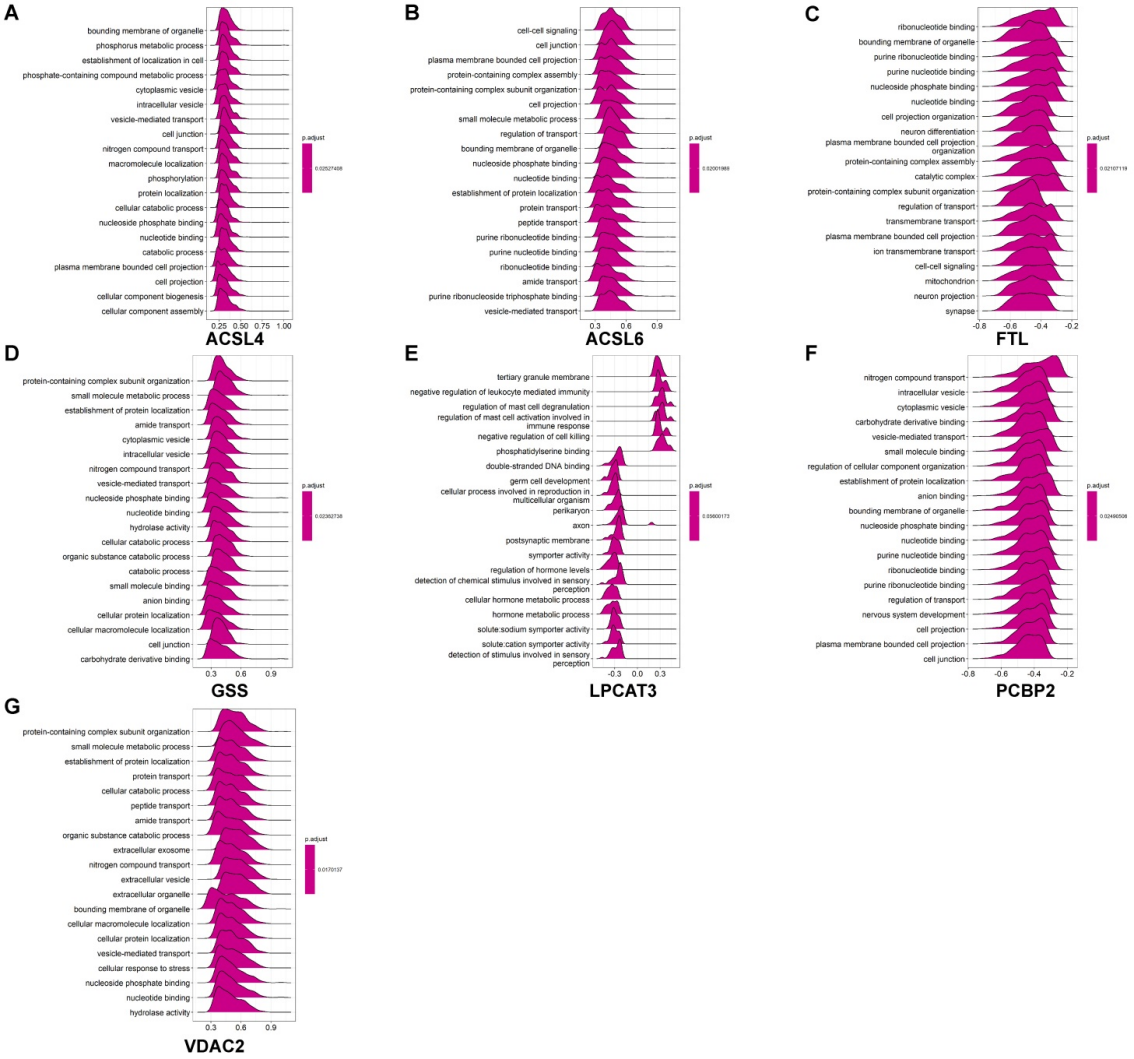
** GO enrichment analysis of coexpressed genes of ferroptosis-related DEGs in AD. (A-G)** GO ridge plot of the top 50 coexpressed genes to *ACSL4*, *ACSL6*, *FTL*, *GSS*, *LPCAT3*, *PCBP2*, and *VDAC2*.

GO analysis revealed that these coexpressed genes regulated different biological functions and pathways (Fig. 6). *ACSL4* is a cellular membrane protein that promotes the ferroptosis process by activating PUFAs for phospholipid biosynthesis 21. The top 50 coexpressed genes of *ACSL4* enriched in the GO categories included bounding membrane of organelle, phosphorus metabolic process, and establishment of cell localization (Fig. 6A). *FTL* encodes ferritin light chain, which is the subunit of a cytosolic iron storage protein. FTL protein can regulate ferroptosis by controlling free iron levels 22. Homozygous loss of function mutation in *FTL* caused a neurodegenerative disease hallmarked by idiopathic generalized seizures and atypical restless leg syndrome 23. The top 50 coexpressed genes of *FTL* enriched in GO terms including ribonucleotide binding, bounding membrane of organelle, and cell projection organization (Fig. 6C).

Figure 6 displays GO enrichment results for other ferroptosis-related DEGs. The coexpressed genes *GSS*, *ACSL6* and *PCBP2* (Figs. 6B, 6D, 6F, respectively) shared some GO terms, including the establishment of protein localization, cell junction, and transport regulation. The GO enrichment on coexpressed gene *VDAC2* exhibited biological processes on extracellular exosomes and extracellular vesicles, whereas some immune-related GO terms (negative regulation of leukocyte mediated immunity and regulation of mast cell activation involved in immune response) are also involved in postsynaptic membrane and axon in *LPCAT3* associated coexpressed genes (Figs. 6E-6G).

**Figure 7:**
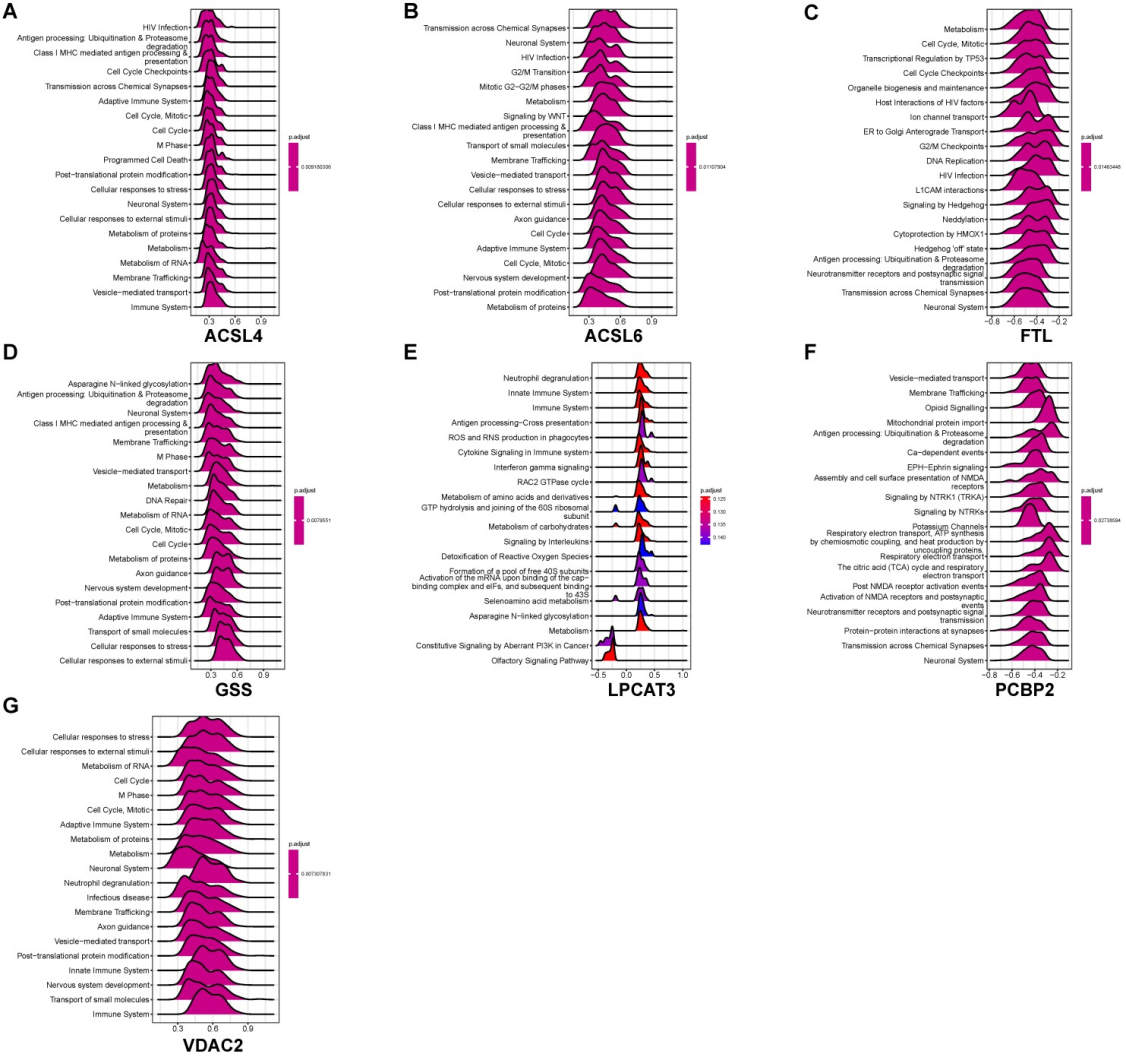
** GSEA identifies coexpression network modules of seven ferroptosis-related DEGs to the REACTOME pathways. (A-G)** GSEA of coexpressed *ACSL4*, *ACSL6*, *FTL*, *GSS*, *LPCAT3*, *PCBP2*, and *VDAC2* to ferroptosis-related DEGs.

We performed GSEA to identify coexpression network modules of seven ferroptosis-related DEGs in relation to REACTOME pathways. Figure 7 depicts the ridge plot of the top 20 REACTOME pathways of seven ferroptosis-related DEGs. The genes participated in pathways closely related to AD pathological mechanism, including ubiquitination and proteasome degradation (*ACSL4*, *GSS*), programmed cell death (*ACSL4*), and cellular responses to stress (*ACSL4*, *ACSL6*, *GSS*, *VDAC2*). Pathways related to the biological function of nervous system, including the neuronal system (*ACSL4*, *ACSL6*, *GSS*, *VDAC2*), neurotransmitter receptors and postsynaptic signal transmission (*FTL*, *PCBP2*, *ACSL4*), nervous system development (*VDAC2*, *GSS*, *ACSL6*), were also affected. Immune pathways were also involved in AD pathology. The REACTOME pathways on seven ferroptosis-related DEGs involved the adaptive immune system (*VDAC2*, *GSS*, *ACSL6*, *ACSL4*), innate immune system (*VDAC2*, *LPCAT3*), and neutrophil degranulation (*VDAC2*, *LPCAT3*). Our results indicate that ferroptosis may directly or indirectly regulate a series of biological processes and pathways, thereby influencing the AD pathogenesis.

### Ferroptosis-related DEGs regulate the immune cell infiltration pattern in the AD hippocampus

**Figure 8:**
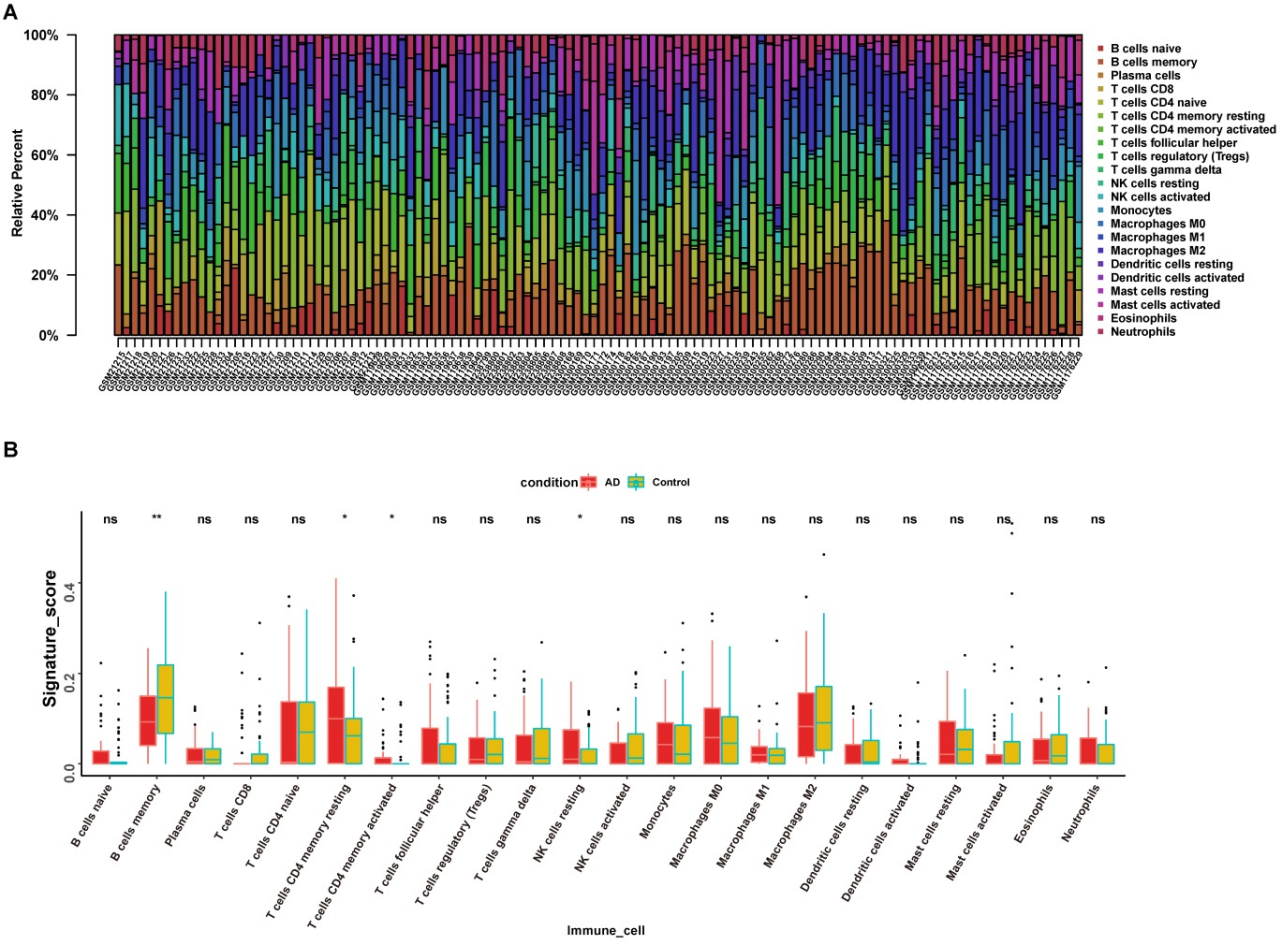
** Immune cell infiltration pattern in AD hippocampus. (A)** Infiltrated immune cell category and proportion in AD and control samples. **(B)** Four types of immune cells had significantly different proportions in AD patients compared with controls. *, *P* < 0.05; ns, not significant.

The infiltration of immune cells and the release of pro-and anti-inflammatory cytokines are crucial to AD pathology 24. Our previous results suggested that *LPCAT3* was down-regulated in the AD hippocampus and could regulate an immune-related biological process. Therefore, we analyzed the infiltrated immune cell category and proportion in our AD and control samples (Fig. 8A), and found that 22 types of immune cells are present among AD and normal hippocampus.

We further calculated infiltration differences of immune cells between AD and control samples among the 22 types of immune cells and found that only 4 types of immune cells had significantly different proportions in patients versus controls (Fig. 8B). Compared with control, AD hippocampus demonstrated decreased memory B cells (0.1005±0.06696 vs 0.1463±0.0961, *P* = 0.0049), increased memory resting CD4^+^ T cells (0.1166±0.1111 vs 0.0720±0.0961, *P* = 0.0123), increased memory activated CD4^+^ T cells (0.016483 ± 0.034499 vs 0.010017 ± 0.029136, *P* = 0.0227), and increased resting NK cells (0.0423 ± 0.0501 vs 0.0347 ± 0.029136, *P* = 0.0099).

**Figure 9:**
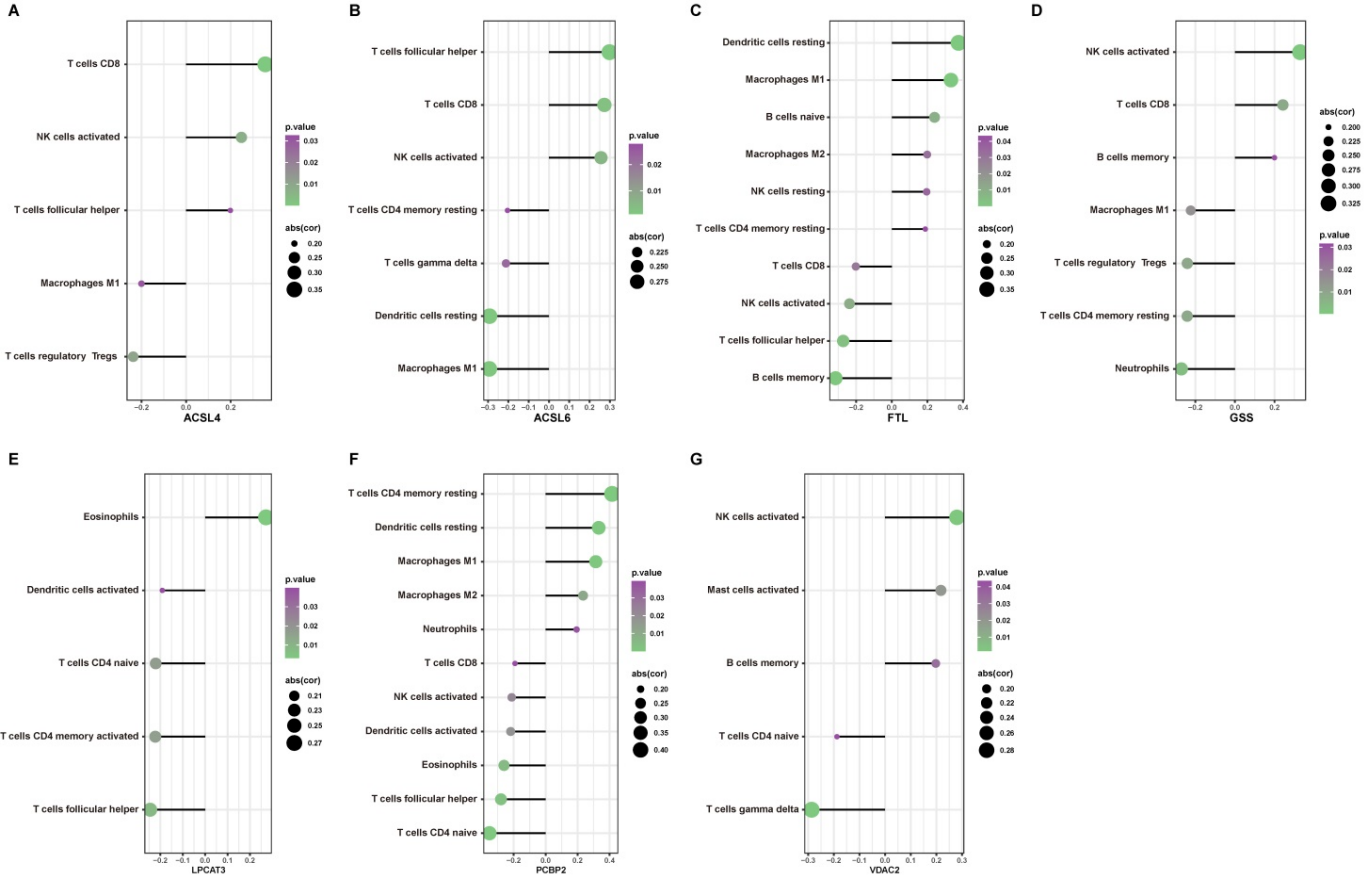
** The correlation between ferroptosis related DEGs and immune cell infiltration. (A-G)** Significant correlations (*P* < 0.05) between infiltrated immune cell types and ferroptosis-related DEGs.

To determine the possible role of ferroptosis in the immune cell infiltration of AD hippocampus, we analyzed the correlation between previously defined ferroptosis-related DEGs and immune cell infiltration. Figure 9 depicts immune cell types with significant correlation. The expression of *ACSL4* positively correlated with the infiltration of CD8^+^ T cells, activated NK cells, and follicular T helper cells, but negatively correlated with the infiltration of M1 macrophages and Tregs (Fig. 9A). The expression of *ACSL6* positively correlated with the infiltration of CD8^+^ T cells, activated NK cells, and follicular T helper cells, but negatively correlated with the infiltration of CD4^+^ memory T cells, γδ T cells, resting dendritic cells, and M1 macrophages (Fig. 9B). The expression of *FTL* positively correlated with the infiltration of resting dendritic cells, M1 macrophages, naive B cells, M2 macrophages, resting NK cells, and resting memory CD4^+^ T cells, but was negatively correlated with CD8^+^ T cells, activated NK cells, follicular T helper cells, and memory B cells (Fig. 9C). The expression of *GSS* positively correlated with the infiltration of activated NK cells, CD8^+^ T cells, and memory B cells, but negatively correlated with neutrophils, resting memory CD4^+^ T cells, Tregs, and M1 macrophages (Fig. 9D). The expression of *LPCAT3* positively correlated with the infiltration of eosinophils, but negatively correlated with activated dendritic cells, naïve CD4^+^ T cells, activated memory CD4^+^ T cells, and follicular T helper cells (Fig. 9E). The expression of *PCBP2* positively correlated with the infiltration of resting memory CD4^+^ T cells, resting dendritic cells, M1 macrophages, M2 macrophages, and neutrophils, but negatively correlated with CD8^+^ T cells, activated NK cells, activated dendritic cells, eosinophils, naïve CD4^+^ T cells, and follicular T helper cells (Fig. 9F). The expression of *VDAC2* positively correlated with the infiltration of activated NK cells, activated mast cells, and memory B cells, but negatively correlated with naïve CD4^+^ T cells and γδ T cells (Fig. 9G). Our findings suggest that the expression changes of ferroptosis in the AD hippocampus may alter specific types of immune cell infiltration.

### Ferroptosis-related DEGs can predict the AD probability

**Figure 10:**
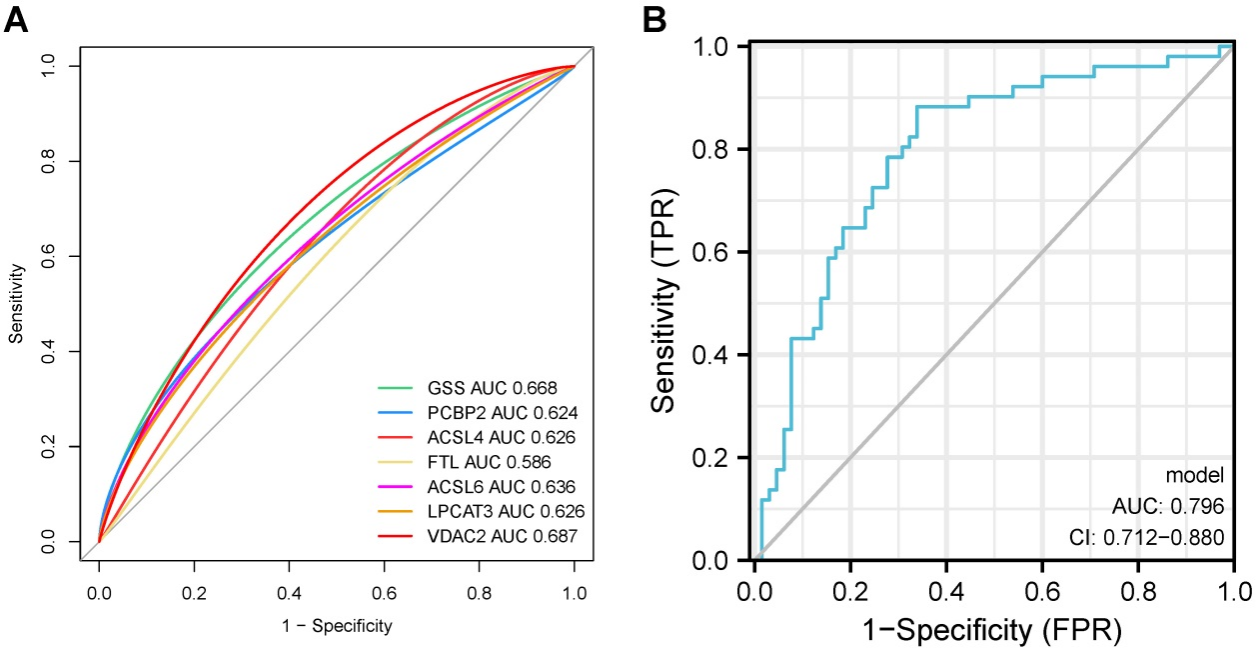
ROC curves of seven ferroptosis-related DEGs on the outcome of AD occurrence in our datasets.

The predictive probabilities generated by the ferroptosis-related DEGs-related diagnostic model were used to generate a single and merged ROC curve. Every single ferroptosis-related DEG demonstrated relatively sufficient diagnostic accuracy with an AUC ranging from 0.586 to 0.687, whereas Fig. 10 displays an excellent diagnostic accuracy with an AUC of 0.796 (CI = 0.712 - 0.880). These results indicate that the model constructed by the seven ferroptosis-related DEGs may predict, to some extent, the outcome of AD occurrence.

### Ferroptosis-related DEGs are altered in the hippocampus and forebrain cortex of AD mice

In our previous results, the ferroptosis-related genes *PCBP2* and *FTL* significantly up-regulated in the hippocampus of AD patients, while *VDAC2*, *LPCAT3*, *GSS*, *ACSL4*, and *ACSL6* significantly down-regulated in AD patients. To further validate the ferroptosis pathway dysregulation in AD, we analyzed the expression of these genes in the brains of APPswe/PSEN1dE9 mice and wildtype mice. Transgenic mice express a chimeric mouse/human amyloid precursor protein (Mo/HuAPP695swe) and a mutant human presenilin 1 (PS1-dE9), both of which are associated with early-onset AD. We performed qPCR on RNA from the hippocampus and forebrain cortex of 6-months-old APPswe/PSEN1dE9 mice and C57BL/6 wildtype mice. As evident in Fig. 10, *Pcbp2* significantly up-regulated in AD hippocampus (1.7740±0.1412, *P* = 0.0056) and forebrain cortex (2.1550±0.0654, *P* = 0.00006), while *FTL* significantly down-regulated in AD hippocampus (1.7740±0.1412, *P* = 0.0056) and forebrain cortex (2.1550±0.0654, P=0.00006). *Gss* was significantly decreased in AD hippocampus (0.1905± 0.1921, *P* = 0.0131) and forebrain cortex (0.3068± 0.0936, *P* = 0.0017). *Acsl4* significantly up-regulated in AD hippocampus (4.415± 0.8115, *P* = 0.0137) and forebrain cortex (3.6830±0.5497, *P* =0.0083), *Acsl6* significantly up-regulated only in AD mice's hippocampus (1.984± 0.8115, *P* = 0.2282) but not in forebrain cortex (1.240± 0.1831, *P* = 0.2760). Moreover, *Vdac2* and *Lpcat3* demonstrated no expression changes in the AD mice's hippocampus or forebrain cortex. Together, our results further support the conception that the AD brain reveals dysregulated ferroptosis gene expression and indicates ferroptosis is a potential therapeutic target for AD.

## Discussion

In this study, we combined hippocampal gene expression array data of 65 controls and 51 AD subjects from the GEO database and identified the DEGs. GO and KEGG analyses were performed on these genes. In the AD hippocampus, *NOTCH2NL*, *TGFB1I1*, and *LTF* up-regulated, while *ARPC1A*, *CHGB*, and *MPV17* down-regulated, indicating that the DEGs identified here can be used as AD pathological markers or potential treatment targets. Recent research has reported that LTF, a member of the transferrin family, can interact with APPww to regulate amyloidogenic processing and neuronal Aβ production, thereby serving as the key predictor of amyloid pathology 25. Moreover, studies have proved that negative regulation of the cell cycle 26, ATP metabolic process 27, cellular respiration 28 and IL1β-mediated signal pathway 29 are involved in AD pathology, which is consistent with our KEGG pathway analysis on the DEGs in AD identified in our study.

AD occurrence is highly related to activated neuronal or peripheral immune inflammation within patients. Compared with controls, the infiltrated immune cell category of AD hippocampus revealed a decrease in memory B cells and an increase in memory resting CD4^+^ T cells, memory-activated CD4^+^ T cells, and resting NK cells. Studies have demonstrated that CD4^+^ T cells, CD8^+^ T cells, resting NK cells, natural killer cells, monocyte-macrophage cells, and activated dendritic cells infiltrated abnormally within AD brain 30 and peripheral blood 31. Moreover, inhibited immune cells could mitigate AD immune dysregulation and cognitive dysfunction. In an AD mouse model, the depletion of NK cells by anti-NK1.1 antibodies drastically alleviate the immune response in the brain, thereby improving cognitive function 32. Therefore, studying the role of immune and inflammatory cells in AD may yield anti-inflammatory and immune regulatory targets for AD treatment.

Iron dyshomeostasis and ferroptosis contribute to the AD progression of 33, indicating that anti-ferroptotic therapies may be effective in AD treatment. Our analyses discovered that nine ferroptosis-related genes significantly altered in the AD hippocampus. Functional interaction and correlation analyses indicated that ferroptosis-related genes are associated with pathways such as protein transportation and localization, extracellular exosome and extracellular vesicle, and immune response. Studies have proved the specific role of ferroptosis in AD. Ferroptosis-related pathological changes have been detected in the brain of AD patients and AD mouse models, including iron metabolic abnormality, glutamate-mediated excitotoxicity, and ROS-caused oxidized lipid accumulation 34. Iron homeostasis disruption and elevated ferritin have been observed in AD patients, along with the increased expression of a light-chain subunit of the cystine/glutamate transporter (xCT) and lipid peroxidation 34.

Ferroptosis may impair both the cognition function and neuronal survival of AD patients. Forebrain and hippocampal neuronal-specific GPX4 KO mice demonstrated apparent cognition dysfunction and hippocampal neuron degeneration, whereas ferroptosis inhibitor Liproxstatin-1 can reverse these AD-like phenotypes 10. Likewise, other ferroptotic inhibitors such as Forsythoside A, can partially mitigate AD pathological phenotype 35, 36. In our AD mice, expressing dysregulation of ferroptosis-related genes can be observed, although the expression of *VDAC2* and *LPCAT3* remain unaltered; *ACSL4* and* ACSL6* down-regulated in AD mice while they up-regulated in patients' hippocampus. These results further support the conception that ferroptosis is dysregulated in the AD brain. These discordances of ferroptosis gene expression between patients and mice may be because the mice mimic specific AD types with a defined genetic background, whereas the etiology of AD patients is often caused by complex genetic and genotype-environment interactions. Together, ferroptosis is strong associated with AD, leading to AD and other neurodegenerative diseases.

The transcriptome study is always a crucial issue in AD mechanism research, as it may contribute to AD onset and progression. For example, transcriptomic analysis through Illumina RNA-Seq on total brain, frontal and temporal lobes of healthy and AD post-mortem tissue revealed an overrepresentation of neuron's cytological structure and synapse function-related genes in AD brain, and alternative splicing and promoter of the *APOE* gene in AD brain is correlated with the progression of neurodegeneration 37. A recent meta-analysis of 2,114 post-mortem AD human brain transcriptomes revealed 30 perturbed coexpression modules enriched in neuronal or microglial genes in mouse models of AD, Huntington's disease, amyotrophic lateral sclerosis, and aging 38. Very few ferroptosis-related transcriptome analyses on the human brain have been reported.

However, several limitations exist in our present study. First, our results shed some light on ferroptosis as an AD cause in patients and mice, but the core ferroptotic genes leading to AD must be clarified, and some inference research should be conducted to explain their mechanism. Moreover, potential gaps exist in the clinical implementation of ferroptosis-related mechanisms for AD treatment, requiring further investigation. For example, whether manipulating specific ferroptosis genes can reserve neural degeneration in AD patients is still unknown, although some ferroptosis inhibitors such as iron chelator DFE and anti-ferroptotic agent vitamin E are undergoing clinical trials 39. Understanding the possible mechanisms behind AD, ferroptosis, and their intersectional relationship provides new insights for AD prevention and treatment.

## Supplementary Material

Supplementary figure and table.

## Figures and Tables

**Figure 11:**
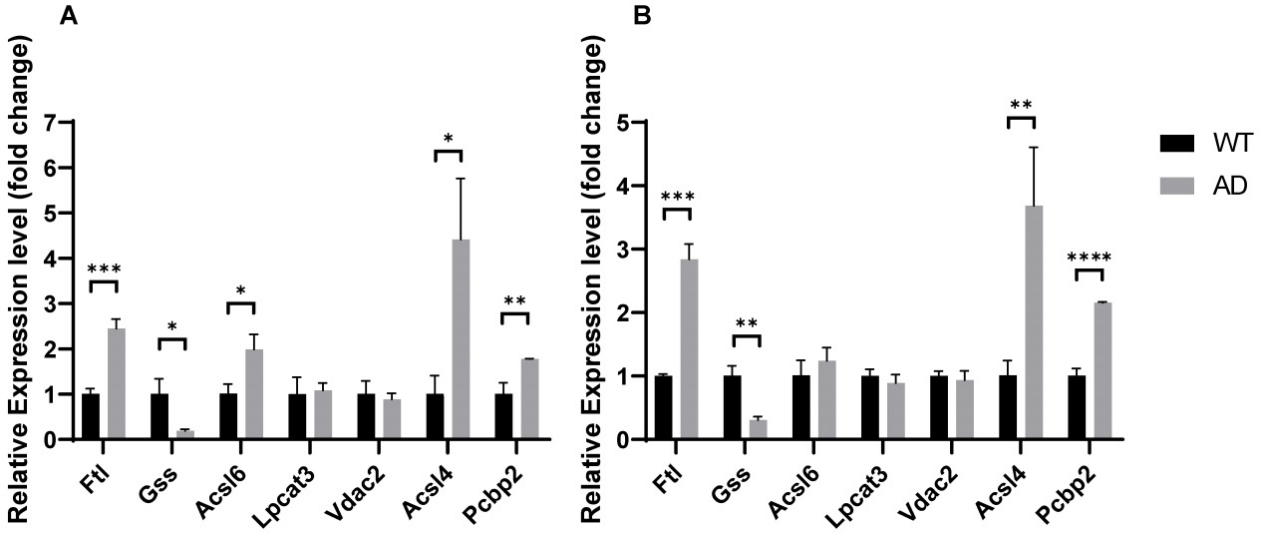
mRNA relative expression level of ferroptosis-related genes in hippocampus and forebrain cortex of AD mice compared with control. *, *P* < 0.05; **, *P* < 0.01; ***, *P* < 0.001; ****, *P* < 0.0001.

**Table 1:** Demographic information between patients and controls

	Control	Patients
Patients No.	65	51
Age (median, range)	79 (20, 102)	85 (60, 101)
**Gender (%)**		
Female	36.9	58.8
Male	63.1	41.2

**Table 2:** Clusters and subnetwork of PPI with a high combined score among the ferroptosis-related DEGs

Rank	Gene	MCC	DMNC	MNC	Degree	EPC	BottleNeck	EcCentricity	Closeness	Radiality	Betweenness	Stress	ClusteringCoefficient
**Top 10 in network 1 ranked by MCC method**													
1	CP	6480	9	9	9	5.172	2	1	9	2.33333	1.83333	10	0.86111
2	SLC11A2	6480	9	9	9	5.164	1	1	9	2.33333	1.83333	10	0.86111
3	SLC40A1	6480	9	9	9	5.274	1	1	9	2.33333	1.83333	10	0.86111
4	TFRC	6480	9	9	9	5.172	2	1	9	2.33333	1.83333	10	0.86111
5	FTH1	5760	8	8	8	4.95	1	0.5	8.5	2.22222	0.66667	4	0.92857
6	FTL	5760	8	8	8	4.948	1	0.5	8.5	2.22222	0.66667	4	0.92857
7	STEAP3	5760	8	8	8	5.03	1	0.5	8.5	2.22222	0.66667	4	0.92857
8	SLC39A14	5760	8	8	8	5.018	1	0.5	8.5	2.22222	0.66667	4	0.92857
9	SLC39A8	720	6	6	6	4.579	1	0.5	7.5	2	0	0	1
10	HMOX1	720	6	6	6	4.335	1	0.5	7.5	2	0	0	1
**Top 3 in network 2 ranked by MCC method**													
1	MAP1LC3B	24	0.56839	4	4	3.357	1	1	4	1.5	0	0	1
2	MAP1LC3A	24	0.56839	4	4	3.348	1	1	4	1.5	0	0	1
3	MAP1LC3C	24	0.56839	4	4	3.324	1	1	4	1.5	0	0	1
**Top 5 in network 3 ranked by MCC method**													
1	ACSL1	24	0.56839	4	4	2.038	1	1	4	1.5	0	0	1
2	ACSL3	24	0.56839	4	4	2.014	1	1	4	1.5	0	0	1
3	ACSL5	24	0.56839	4	4	2.016	1	1	4	1.5	0	0	1
4	ACSL4	24	0.56839	4	4	2.042	1	1	4	1.5	0	0	1
5	ACSL6	24	0.56839	4	4	2.03	1	1	4	1.5	0	0	1

## Abbreviations

AD: Alzheimer's Disease
APP: amyloid precursor protein
PSEN1: presenilin 1
PSEN2: presenilin 2
Aβ: β-amyloid
phospholipid hydroperoxides: PLOOHs
TDP-43: TAR DNA-binding protein 43
differential expressed genes: DEGs
ROS: reactive oxygen species
PUFA-PLs: polyunsaturated fatty acid chains
PUFAs: polyunsaturated fatty acids
NCBI: the National Center of Biotechnology Information
GO: Gene Ontology
KEGG: Kyoto Encyclopedia of Genes and Genomes
Protein-Protein Interactions: PPI
GSEA: Gene Set Enrichment Analysis
operating characteristic curve: ROC
area under the curve: AUC
light-chain subunit of the cystine/glutamate transporter: xCT
