# Supplementary material for: A Bioinformatics Perspective on the Dysregulation of Ferroptosis and Ferroptosis-related Immune Cell Infiltration in Alzheimer's Disease
Source: Int J Med Sci. 2022 Oct 24;19(13):1888–902. doi: 10.7150/ijms.76660 (PMC9682502; doi:10.7150/ijms.76660)
Supplement: Supplementary file 1 — Supplementary figure and table. [file ijmsv19p1888s1.pdf]

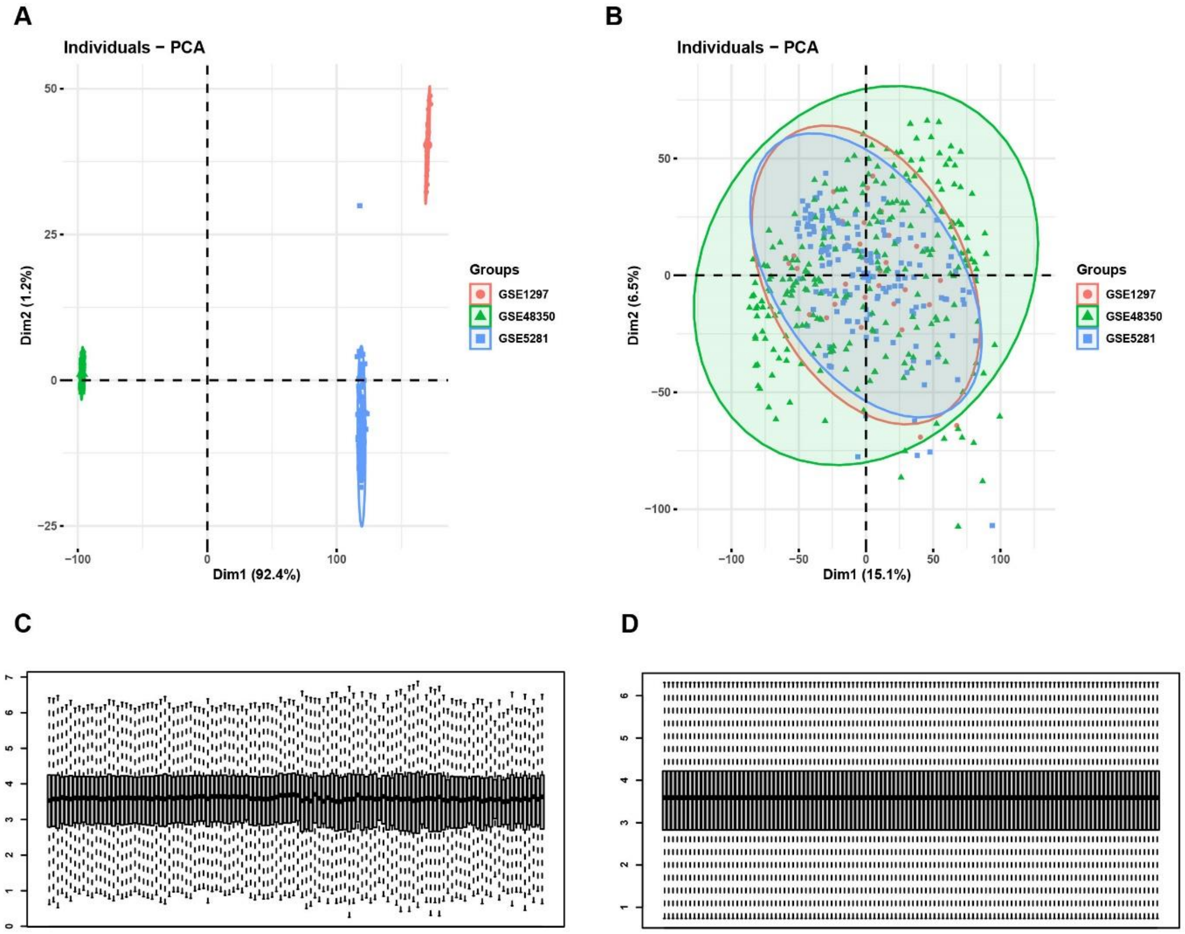

Supplementary Figure 1 Data combination and normalization were successfully performed on 3 separate GEO datasets. (A, B) PCA plot reveals the effect of data before (A) and after (B) batch effect removal. (C, D) data from different datasets have been normalization.

Supplement table 1 KEGG analysis identified 38 significant enriched pathways in AD hippocampus compared with control hippocampus.

ID	Description	GeneRatio	BgRatio	pvalue	p.adjust	qvalue	Count
hsa05016	Huntington disease	57/580	306/9180	6.19E-14	1.93E-11	1.45E-11	57
hsa05020	Prion disease	50/580	273/9180	4.60E-12	7.15E-10	5.40E-10	50
hsa05012	Parkinson disease	48/580	266/9180	2.19E-11	2.27E-09	1.71E-09	48
hsa05014	Amyotrophic lateral sclerosis	58/580	365/9180	3.71E-11	2.88E-09	2.17E-09	58
hsa05010	Alzheimer disease	58/580	384/9180	2.92E-10	1.81E-08	1.37E-08	58
hsa05208	Chemical carcinogenesis - reactive oxygen species	39/580	223/9180	4.51E-09	2.34E-07	1.76E-07	39
hsa03050	Proteasome	16/580	46/9180	8.80E-09	3.91E-07	2.95E-07	16
hsa05022	Pathways of neurodegeneration - multiple diseases	63/580	476/9180	1.09E-08	4.25E-07	3.21E-07	63
hsa00190	Oxidative phosphorylation	27/580	134/9180	5.60E-08	1.93E-06	1.46E-06	27
hsa04932	Non-alcoholic fatty liver disease	28/580	155/9180	3.56E-07	1.11E-05	8.35E-06	28
hsa05017	Spinocerebellar ataxia	26/580	143/9180	8.20E-07	2.32E-05	1.75E-05	26
hsa04218	Cellular senescence	26/580	156/9180	4.47E-06	0.000116	8.75E-05	26
hsa05415	Diabetic cardiomyopathy	30/580	203/9180	1.04E-05	0.000249	0.000188	30
hsa04068	FoxO signaling pathway	22/580	131/9180	2.14E-05	0.000466	0.000352	22
hsa04714	Thermogenesis	32/580	232/9180	2.25E-05	0.000466	0.000352	32
hsa05215	Prostate cancer	18/580	97/9180	3.03E-05	0.000589	0.000444	18
hsa04066	HIF-1 signaling pathway	18/580	109/9180	0.000148	0.002704	0.002041	18
hsa04919	Thyroid hormone signaling pathway	19/580	121/9180	0.000194	0.003346	0.002525	19
hsa05120	Epithelial cell signaling in Helicobacter pylori infection	13/580	70/9180	0.000376	0.00616	0.00465	13
hsa05165	Human papillomavirus infection	37/580	331/9180	0.000473	0.007361	0.005556	37
hsa04015	Rap1 signaling pathway	26/580	210/9180	0.000727	0.010601	0.008001	26
hsa04110	Cell cycle	18/580	124/9180	0.00075	0.010601	0.008001	18
hsa05169	Epstein-Barr virus infection	25/580	202/9180	0.000919	0.012428	0.00938	25
hsa04723	Retrograde endocannabinoid signaling	20/580	148/9180	0.000988	0.0128	0.009661	20
hsa05167	Kaposi sarcoma-associated herpesvirus infection	24/580	194/9180	0.001162	0.014452	0.010908	24
hsa05132	Salmonella infection	28/580	249/9180	0.002057	0.024607	0.018573	28
hsa05161	Hepatitis B	20/580	162/9180	0.002974	0.033489	0.025277	20
hsa04151	PI3K-Akt signaling pathway	36/580	354/9180	0.003015	0.033489	0.025277	36
hsa01522	Endocrine resistance	14/580	98/9180	0.003264	0.033664	0.025409	14
hsa04010	MAPK signaling pathway	31/580	294/9180	0.003339	0.033664	0.025409	31
hsa04540	Gap junction	13/580	88/9180	0.003356	0.033664	0.025409	13
hsa05219	Bladder cancer	8/580	41/9180	0.00361	0.034424	0.025983	8

hsa05211	Renal cell carcinoma	11/580	69/9180	0.00372	0.034424	0.025983	11
hsa01521	EGFR tyrosine kinase inhibitor resistance	12/580	79/9180	0.003763	0.034424	0.025983	12
hsa00061	Fatty acid biosynthesis	5/580	18/9180	0.004254	0.037803	0.028533	5
hsa04213	Longevity regulating pathway - multiple species	10/580	62/9180	0.005112	0.04367	0.032961	10
hsa05218	Melanoma	11/580	72/9180	0.005195	0.04367	0.032961	11
hsa04014	Ras signaling pathway	25/580	232/9180	0.006063	0.049624	0.037455	25
